# Fabrication and Characterization of Nanoporous Niobia, and Nanotubular Tantala, Titania and Zirconia via Anodization

**DOI:** 10.3390/jfb6020153

**Published:** 2015-03-31

**Authors:** Sepideh Minagar, Christopher C. Berndt, Cuie Wen

**Affiliations:** 1 Faculty of Science, Engineering and Technology, Swinburne University of Technology, Hawthorn, Victoria 3122, Australia; E-Mails: sminagar@swin.edu.au (S.M.); cberndt@swin.edu.au (C.B.); 2 Department of Materials Science and Engineering, Stony Brook University, Stony Brook, New York, NY 11794, USA; 3 School of Aerospace, Mechanical and Manufacturing Engineering, RMIT University, Bundoora, Victoria 3083, Australia

**Keywords:** nanotube, tantala, niobia, zirconia, titania, wettability, roughness, hydroxyapatite

## Abstract

Valve metals such as titanium (Ti), zirconium (Zr), niobium (Nb) and tantalum (Ta) that confer a stable oxide layer on their surfaces are commonly used as implant materials or alloying elements for titanium-based implants, due to their exceptional high corrosion resistance and excellent biocompatibility. The aim of this study was to investigate the bioactivity of the nanostructures of tantala (Ta_2_O_5_), niobia (Nb_2_O_5_), zirconia (ZrO_2_) and titania (TiO_2_) in accordance to their roughness and wettability. Therefore, four kinds of metal oxide nanoporous and nanotubular Ta_2_O_5_, Nb_2_O_5_, ZrO_2_ and TiO_2_ were fabricated via anodization. The nanosize distribution, morphology and the physical and chemical properties of the nanolayers and their surface energies and bioactivities were investigated using SEM-EDS, X-ray diffraction (XRD) analysis and 3D profilometer. It was found that the nanoporous Ta_2_O_5_ exhibited an irregular porous structure, high roughness and high surface energy as compared to bare tantalum metal; and exhibited the most superior bioactivity after annealing among the four kinds of nanoporous structures. The nanoporous Nb_2_O_5_ showed a uniform porous structure and low roughness, but no bioactivity before annealing. Overall, the nanoporous and nanotubular layers of Ta_2_O_5_, Nb_2_O_5_, ZrO_2_ and TiO_2_ demonstrated promising potential for enhanced bioactivity to improve their biomedical application alone or to improve the usage in other biocompatible metal implants.

## 1. Introduction

Metals and alloys that are used as biomaterials also have a range of applications in industry and medicine due to their excellent mechanical, physical and chemical properties. Extensive studies have been carried out on titanium that has been coated by its natural oxide layer formed in air. Other biocompatible metals and their respective oxide layers are also of interest. For example, the protective oxide layer that forms naturally on a tantalum surface or that is fabricated on the surface of other metals by surface treatments such as chemical vapor deposition [1], electro deposition [2,3] and sol gel [4] methods have other applications. Tantalum pentoxide, Ta_2_O_5_, is used as a protective coating for chemical equipment due to its excellent corrosion resistance [1]. A thin film of Ta_2_O_5_, formed by radio frequency (RF) sputtering of SiO_2_-Ta_2_O_5_ films, has been used in optical devices and memory devices [5]. The biocompatibility of the tantalum pentoxide layer suggests tantalum as a good candidate for bioengineering implant applications [6]. By anodization a nanoporous Ta_2_O_5_ can be formed on the metal in an acidic electrolyte containing hydrofluoric acid (HF) [7,8,9]. The organic electrolytes were also used for anodizing a nanoporous Ta_2_O_5_ layer at an applied potential of 10–40 V [10,11].

The application of niobium pentoxide (Nb_2_O_5_), which can be produced via different methods, such as reactive sputtering [12], sol-gel processes [13], templating techniques [14] and anodic oxidation [15,16], has been reported in applications for gas sensors [17], catalysts [18], both optical and electrochromic devices [12], solid state electrochemical devices [19] and biocompatible prostheses [13]. There are studies on the formation of a nanoporous layer of Nb_2_O_5_ by controlling the effects of mixed electrolytes, applied potential and anodization time. These nanoporous layers displayed different range of pore sizes and thickness formed mostly in an acidic electrolyte [15,20,21]. An important application for zirconium dioxide (ZrO_2_) is as an industrial catalyst, especially with its use as an acid catalyst and for NO*_x_* reduction because it is more stable under hydrothermal conditions in comparison to zeolites. This metal oxide also exhibits large pores and a flexible mix in metal oxide composition [22,23]. Zirconia, ZrO_2_, can have optoelectronic and biomedical applications due to its high mechanical, chemical, and thermal stability [24]. The formation of self-organized sponge-like porous ZrO_2_ [25] and nanotube oxide layer with different nanoscale sizes and thicknesses [26] has been described in detail along with the effect of changing the condition of electrolyte, applied potential and time of anodization [26,27,28,29,30].

The composition of the substantial mineral form of hard tissue such as bone and dentin is apatite, which is secreted by bone cells on the implant surface during the process of attachment. The bioactive materials, which contained Si–OH, Ti–OH, Zr–OH, Nb–OH and Ta–OH, have been reported to exhibit the ability to induce apatite formation [31]. The effect of Ta–OH groups on the surface of alkaline treated bioactive tantalum metal to form bone like apatite was investigated and the Ca/P ratio of 1.59 was reported for crystalline apatite after immersing in simulated body fluid (SBF) [32]. The nanoporous niobium oxides, which were prepared by sol-gel method and coated on 316LSS enhanced hydroxyapatite formation [33]. Nanosheets and nanofiber-like surface formed on niobium surface by hydrothermal alkaline treatment significantly influenced its apatite inducing ability [34]. The porous tantalum coating surface was reported to enhance osseoinductivity *in vitro* and promote new bone formation *in vivo* [35]. Amorphous nanoporous bioactive sodium niobate hydrogel layer formed on the surface of niobium by alkaline treatment induced the deposition of a CaP layer during soaking in SBF [36]. As formed ZrO_2_ nanotubes have been reported to induce apatite formation [37], and annealing improved its bioactivity [38]. Using pre-treatment, such as effective dipping treatment, improved bioactivity of as formed ZrO_2_ nanotube with diameter 35 to 80 nm [39]. The osseointegration was observed for the anodized surface of zirconium implanted in Wistar rats [40]. Although a few studies have investigated the bioactivity of ZrO_2_ nanotubes, there is still a lack of research in this aspect for nanoporous Nb_2_O_5_ and Ta_2_O_5_, which is the object of this study.

In this study, a close observation has been carried out on the relationship between the nanoporous and nanotubular characteristics of Ta_2_O_5_, Nb_2_O_5_, ZrO_2_ and TiO_2_ such as roughness, wettability and surface energy and the bioactivity. The bioactive property of the surface layers is important in terms of the biomedical applications of these biocompatible metals. However, there are insufficient studies in this aspect, to date. The prime motivation for the current work is to elucidate the influence of the nano characteristics of the surface layer on the bioactivity of the substrate metals.

## 2. Results and Discussion

### 2.1. The Dynamics of the Anodization Process for Tantala (Ta_2_O_5_), Niobia (Nb_2_O_5_) and Zirconia (ZrO_2_)

**Figure 1:**
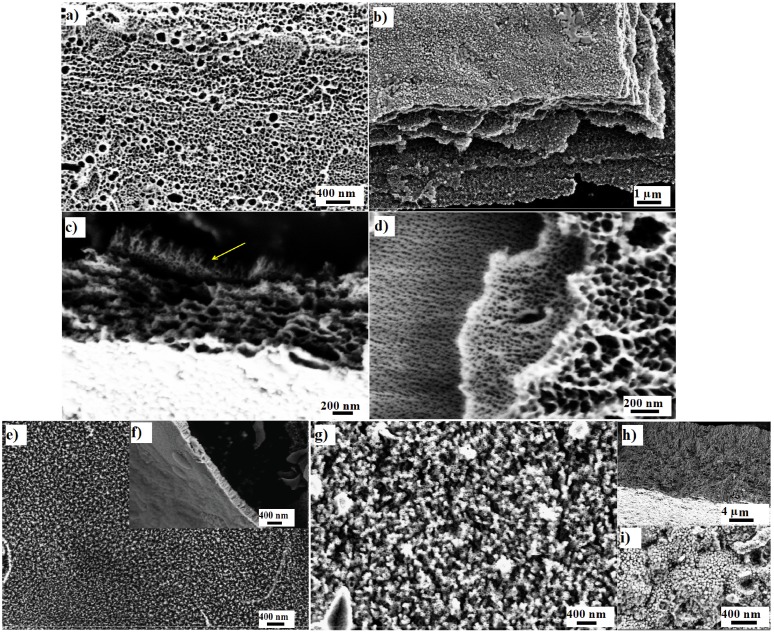
SEM image of nanoporous Ta_2_O_5_: (**a**) top view; (**b**) the bottom view and the cross section of multilayer; (**c**) shorted life nanotubes (arrow); (**d**) the top view of multilayer formed on a Ta after anodization for 120 min in 1 M H_2_SO_4_ + 3.3 wt % NH_4_F, 20 V, SEM image of nanoporous Nb_2_O_5_; (**e**) top view and (**f**) cross section formed on niobium after anodization for 16 min in 1 M H_2_SO_4_ + 3.3 wt % NH_4_F, 20 V and SEM image of ZrO_2_ nanotubes (**g**) top view; (**h**) cross section and (**i**) bottom view formed on a zirconium after anodization for 95 min in 1 M (NH_4_)_2_SO_4_ + 0.3 wt % NH_4_F, pH = 5 and 20 V.

**Figure 2:**
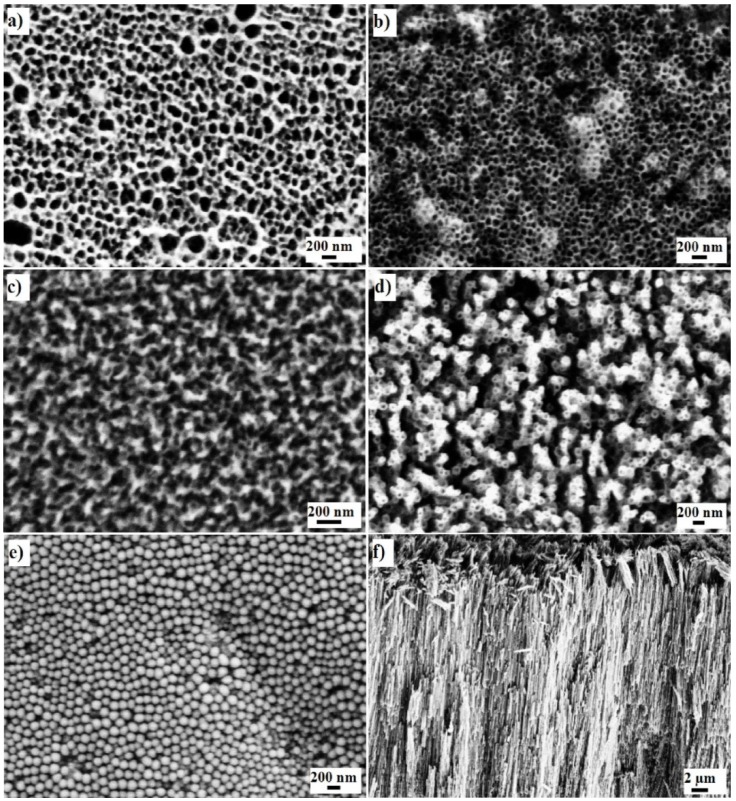
SEM images of nanoporous Ta_2_O_5_: (**a**) top view; (**b**) the middle of the layer; (**c**) nanoporous Nb_2_O_5_; (**d**) top view; (**e**) bottom view; (**f**) cross section of ZrO_2_ nanotubes.

Figure 1a and Figure 2a show a top view of a nanoporous Ta_2_O_5_ that exhibits irregularly distributed pores with a diameter range of 35–65 nm. The thickness of this layer was 1.17 ± 0.05 μm, taking note that the sub-structure revealed multiple layers (Figure 1b and Figure 2b), as reported previously [11], where every layer exhibited a closed bottom. Figure 1c shows the cross section of the multiple layers and the arrow indicates Ta_2_O_5_ nanotubes with a length of nearly 152 ± 1 nm that formed initially after several seconds during anodization. These long nanotubes transformed into nanoporosity after several seconds during anodization and exhibited similar morphological features to other layers. A top view of the multiple layers can be observed in Figure 1d.

Similar to the anodization of Ti as described previously [41], the anodization of Ta, Nb and Zr in the presence of F^−^ was observed in three steps: (i) formation of compact and protective metal oxide with a decay of current density (*vs.* time); (ii) chemical dissolution of the oxide in the presence of the fluoride anion and formation of metal oxide simultaneously with a rise of current density (*vs.* time); and (iii) reaching equilibrium between oxidation and dissolution with a steady state of current density (*vs.* time). The anodization conditions, such as applied potential and concentration of F^−^, were reported to have an influence on the shape of current density *vs.* time curve [7,8,15,20,25,26]. When anodization starts, a compact oxide layer forms on the surface of tantalum for a finite thickness according to the decay characteristics of the current. Tantalum ions, Ta^5+^, arrive at the interface of the oxide layer and electrolyte and become soluble in the electrolyte by forming [TaF_7_]^2−^. Thus, pores formed at the interface between the oxide layer and electrolyte. These pores continued to develop during anodization because they preferred sites that trap the necessary ions. Then, the growth rate of the metal oxide nanoporous and nanotubes may be determined by the diffusion rate of F^−^ ions and the soluble metal complex [42].

Although the formation mechanism for the nanoporosity of tantalum and other valve metals is uncertain, the nanoporosity could be related to the molecular dimension of [TaF_7_]^2−^ and the formation kinetics of Ta_2_O_5_. The standard electrode potential of Ta → Ta^5+^ is 0.75 V, which is less than that of Ti → Ti^4+^, 2.132 V [43]. Thus, Ta^5+^ oxidizes quickly. In addition, the standard enthalpies of formation for Ta_2_O_5_ and TiO_2_ are −492.790 and −228.360 gram calories per mole, respectively [44]. It can be concluded that the oxidization process and the formation of a Ta_2_O_5_ layer on tantalum are faster than the processes on the titanium surface.

The mechanism and kinetics for the oxide layer formation can be calculated on the basis of the ionic radii for Ta^5+^ and F^−^ on a first principles basis. In this instance, the physical and chemical environment around the [TaF_7_]^2−^ at the pore sites, which affect the molecular size of [TaF_7_]^2−^, are ignored. The structure of the complex has been reported to be a two pentagonal pyramid. The volume of the circumscribed sphere of this structure can be calculated as 26.094 × 10^−30^ m^3^, which is smaller than the octahedral volume of [TiF_6_]^2−^ = 29.182 × 10^−30^ m^3^. It can be expected that the diffusion of [TaF_7_]^2−^ is faster than [TiF_6_]^2−^ and that the pore growth does not occur solely at the bottom, but also at the walls, thereby resulting in pores that merge and agglomerate. Over a period of several seconds, the pore was bottle-shaped, similar to that of nanotubes with a closed bottom where the diameter of tubes near the bottom was greater than that at the tube necks. Gaps are also created at the bottom of the layer when the pores merged, which permits the electrolyte to become available underneath the nanotubes. Thus, a new layer of nanotubes can form under the same mechanism, thereby resulting in the formation of multilayers of nanoporous layer over time. The nanotubes dissolve according to the dissolution rate of the oxide layer in the presence of the fluorine ions (F^−^). 

The nanoporous Nb_2_O_5_ (as shown in Figure 1e and Figure 2c) exhibited nearly uniform pore diameters of 22–42 nm. The thickness of this layer was 242 ± 33 nm (Figure 1f). The same formation mechanism can be hypothesized for nanoporous Nb_2_O_5_ layer formation as has been described for the formation of the nanoporous Ta_2_O_5_ layer. Niobium (Nb) and Zr are in the same period and adjacent columns in periodic table of the elements. The standard electrode potential of Nb → Nb^5+^ is 0.644 V, which is less than that of Zr → Zr^4+^, 1.553 V [43]; Nb is, therefore, oxidized rapidly. In addition, the standard enthalpy of formation for Nb_2_O_5_ is −458.640 gram calories per mole, whereas the enthalpy of formation is −264.199 gram calories per mole for ZrO_2_ [44].

The processes of oxidization and formation of the oxide layer on the niobium surface are more rapid than those on the zirconium surface. Consider, for example, that the physical and chemical environment around the [NbF_7_]^2−^ ion within the pore structure are ignored. The volume of the ion complexes can then be calculated on the basis of the ionic radii for Nb^5+^ and F^−^ ions and assuming a lattice structure of a two pentagonal pyramid. The volume of the circumscribed sphere of the [NbF_7_]^2−^ ion is calculated as 32.02 × 10^−30^ m^3^, which is smaller than the volume of the [ZrF_6_]^2−^ ion, which is 36.08 × 10^−30^ m^3^, which is octahedral. It can be expected, therefore, that the diffusion of a [NbF_7_]^2−^ ion within a pore is more rapid than for a [ZrF_6_]^2−^ ion. The growth of the pore does not arise only at the bottom but also at the walls, which will result in merging of pores. 

Figure 1g–i and Figure 2d–f show the fabricated ZrO_2_ nanotubes that exhibit: (i) uniform inner pore diameters of 21–35 nm; (ii) an outer diameter of 54–68 nm; and (iii) wall thickness of 10–16 nm. The thickness of this layer was about 24.52 ± 0.74 μm. As previously explained [45], the standard electrode potential of Zr → Zr^4+^, 1.553 V, is lower than that of Ti → Ti^4+^, 2.132 V [43], which results in rapid oxidation. In addition, the standard enthalpy of formation for ZrO_2_ is −264.199 gram calories per mole, whereas that for TiO_2_ is −228.360 gram calories per mole [44]. The process of oxidation and the formation of the oxide layer on the surface of zirconium are faster than those for the titanium surface. However, the formation of the [ZrF_6_]^2−^ ion takes more time than for the [TiF_6_]^2−^ ion because the solubility equilibrium constant of (NH_4_)_2_ZrF_6_ is higher than that of (NH_4_)_2_TiF_6_. Based on the hypothesis described previously, the volume of the circumscribed sphere of the octahedral structure of [ZrF_6_]^2−^ can be calculated as 36.08 × 10^−30^ m^3^ and 29.18 × 10^−30^ m^3^ for [TiF_6_]^2−^, which causes a lower diffusion rate inside the pore. It has been reported that nanotubes grow longer with a smaller diameter when the dissolution rate is slow compared to the oxidation process [46]. 

### 2.2. Physical Characteristics of Nanoporous Ta_2_O_5_ and Nb_2_O_5_ and Nanotubular ZrO_2_ Layers

**Figure 3:**
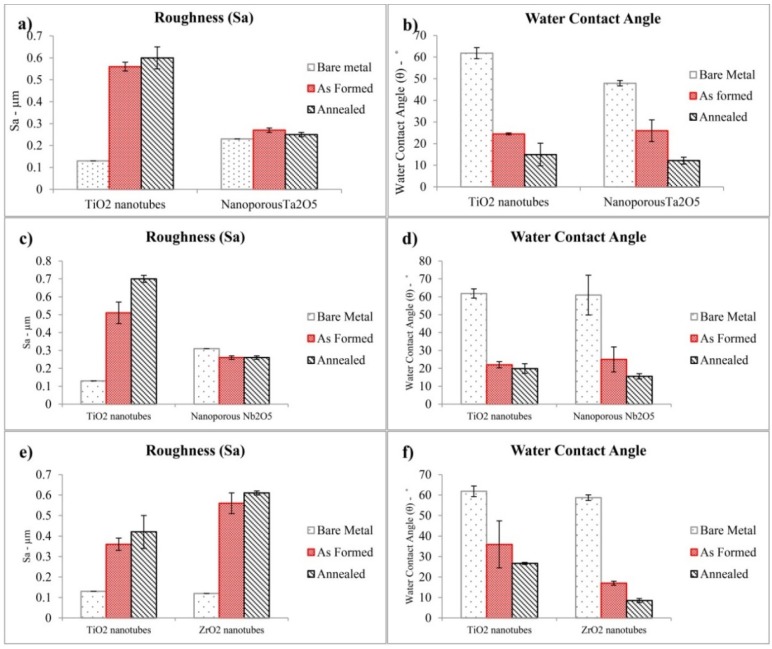
Demonstration of (**a**,**c**,**e**) changes of roughness (*S*_a_) and (**b**,**d**,**f**) modification of water contact angle of bare tantalum, niobium, zirconium and titanium after fabrication of nanotubular and nanoporous layer. Each data point is an average of three measurements.

Figure 3a demonstrates the influence of the formation of nanoporous Ta_2_O_5_ and the TiO_2_ nanotubes on the *S*_a_ with respect to the bare metals. The nanoporous layer of Ta_2_O_5_ revealed a lower surface roughness than the TiO_2_ nanotubes, with almost the same distribution of inner diameter (*D_i_*), when the concentration of fluoride ion $\left(C_{F}^{-}\right)$ was 0.5 wt % at 20 V. The roughness similarity arose because the TiO_2_ nanotubes were separated from each other, whereas the nanoporous Ta_2_O_5_ formed a continuous layer. The surface area index (*S*_I_) may be calculated by dividing the projected surface area, namely, the total exposed three-dimensional surface area being analyzed, including peaks and valleys, to the surface area measured in the lateral direction. The volume index (*V*_I_) may be calculated by dividing the natural volume, namely, the amount of liquid that it would take to submerge the dataset to its highest point, to the normal volume that is measured in the lateral of the nanoporous layer. The *S*_I_ and *V*_I_ values of nanoporous Ta_2_O_5_ are shown in Table 1, with the corresponding roughness amplitude parameters, and verify that the Ta_2_O_5_ nanoporous layer did not significantly change the surface area and roughness of the bare metal. Although the distribution of nanoporous Ta_2_O_5_ was not uniform, there were no high peaks and low valleys according to the measured *S*_skw_, which is close to zero. The peaks and valleys exhibited a platykurtic distribution that was almost uniform due to the *S*_ku_ being near to 3.

**Figure 4:**
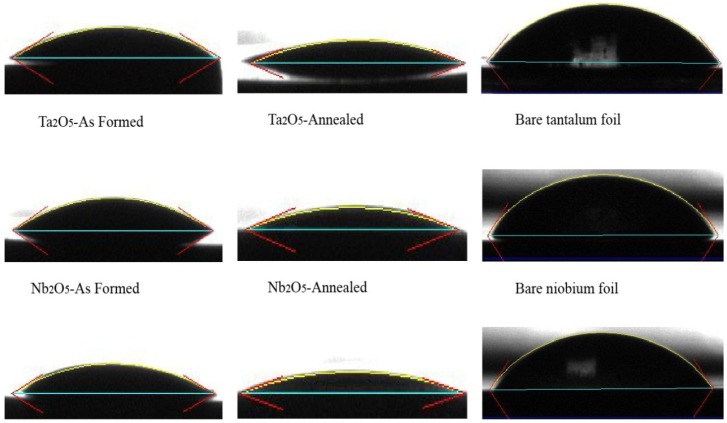
Photo images of water droplet on the surface of nanoporous and nanotubular layers.

Figure 3b shows the change of hydrophilic properties of bare tantalum and titanium after fabrication of the nanoporous and nanotubular layers on their surfaces. The water contact angle measurement of a surface represents the wetting properties of the surface. The literature defines a surface as superhydrophobic when the water contact angle (θ_w_) is more than 150° and hydrophobic when it is 90° < θ_w_ < 150°. A surface has superhydrophilic properties when θ_w_ < 10° and has hydrophilic properties with a water contact angle 10° < θ_w_ < 90° [47]. The as-formed nanoporous Ta_2_O_5_ exhibited hydrophilic properties that were similar to the TiO_2_ nanotubes with the same distribution of inner diameters, *D_i_* (under the conditions of $C_{F}^{-}$ = 0.5 wt %, 20 V). Figure 4 presents photo images of a water droplet on the surface of nanoporous and nanotubular layers. Figure 3a,b also suggests a direct relationship between roughness and water contact angle. When the roughness increased there was a decrease in the water contact angle. The calculated surface energy for nanoporous Ta_2_O_5_ increased after anodization and also after annealing due to the completion of its crystallization. The water contact angle and surface energy have been detailed in Table 2 for nanoporous Ta_2_O_5_.

**Table 1:** Surface area and volume index and roughness amplitude parameters of nanoporous Ta_2_O_5_, Nb_2_O_5_ and nanotube ZrO_2_. Each data point is an average of three measurements.

Material	Surface area index (*S*_I_)	Volume index (*V*_I_)	*S*_a_(μm)	*S*_q_(μm)	*S*_skw_	*S*_ku_
Nanoporous Ta_2_O_5_; As formed	1.34 ± 0.01	18393 ± 86	0.27 ± 0.01	0.35 ± 0.01	0.60 ± 0.23	4.82 ± 0.52
Nanoporous Ta_2_O_5_; Annealed (10 min at 290 °C)	1.27 ± 0.01	1838 ± 132	0.25 ± 0.01	0.32 ± 0.01	0.72 ± 0.03	3.90 ± 0.17
Bare tantalum foil (0.1 mm)	1.06 ± 0.00	18229 ± 0	0.23 ± 0.00	0.29 ± 0.00	0.06 ± 0.00	2.79 ± 0.00
Nanoporous Nb_2_O_5_; As formed	1.50 ± 0.00	18398 ± 134	0.26 ± 0.00	0.34 ± 0.00	0.64 ± 0.03	3.78 ± 0.21
Nanoporous Nb_2_O_5_; Annealed (10 min at 290 °C)	1.38 ± 0.00	18357 ± 144	0.26 ± 0.01	0.33 ± 0.01	0.39 ± 0.02	3.28 ± 0.05
Bare niobium foil (0.05 mm)	1.04 ± 0.00	18327 ± 0	0.31 ± 0.00	0.38 ± 0.00	0.22 ± 0.00	2.68 ± 0.00
ZrO_2_ Nanotube; As formed	2.44 ± 0.30	18422 ± 12	0.56 ± 0.05	0.71 ± 0.05	−0.86 ± 0.018	3.75 ± 0.49
ZrO_2_ Nanotube; Annealed (10 min at 290 °C)	2.94 ± 0.15	18428 ± 79	0.61 ± 0.01	0.76 ± 0.00	−0.57 ± 0.20	3.30 ± 0.35
Bare zirconium foil (0.05 mm)	1.03 ± 0.00	18531 ± 0.00	0.12 ± 0.00	0.20 ± 0.00	−4.27 ± 0.00	39.31 ± 0.00

Figure 3c indicates the progression in roughness for the nanoporous Nb_2_O_5_ layer and TiO_2_ nanotubes while exhibiting almost the same distribution of *D_i_* (under the conditions of $C_{F}^{-}$ = 0.5 wt %, 15 V) with respect to the corresponding bare metals. The roughness of the titanium increased upon the creation and separation of TiO_2_ nanotubes that give rise to gaps in the surface architecture, whereas the roughness of the niobium decreased upon the growth of nanoporous Nb_2_O_5_. The *S*_skw_ of the Nb_2_O_5_ nanoporous surface is higher than zero, indicating the presence of high peaks and low valleys with a leptokurtic distribution. The surface area and volume indices of nanoporous Nb_2_O_5_ are shown in Table 1, as well as the corresponding roughness amplitude parameters. Figure 3d shows the changes of hydrophilic properties of the bare niobium and titanium after fabrication of the nanoporous and nanotubular layers on their surfaces. The nanoporous surface revealed a water contact angle in the range of almost the same distribution of *D_i_* (under the conditions of $C_{F}^{-}$ = 0.5 wt %, 15 V) for TiO_2_ nanotubes that showed hydrophilic properties. This data also implies that there is a relationship between roughness and the water contact angle. The surface energy of this layer was higher than bare niobium. The water contact angle and surface energy are listed in Table 2 for the nanoporous Nb_2_O_5_.

**Table 2:** Water contact angle and surface energy of as formed and annealed nanoporous Ta_2_O_5_, Nb_2_O_5_ and nanotube ZrO_2_ in comparison to its bare metal. Each data point is an average of three measurements.

Material	Contact angle (θ/°)	$\gamma_{S}^{d}$ (mJ·m^−2^)	$\gamma_{S}^{p}$ (mJ·m^−2^)	$\gamma_{S}=\gamma_{S}^{d}+\gamma_{S}^{p}$ (mJ·m^−2^)
Nanoporous Ta_2_O_5_; As formed	26.0 ± 5.0	11.8	55.2	67.0
Nanoporous Ta_2_O_5_; Annealed (10 min at 290 °C)	12.1 ± 1.6	15.2	56.7	71.9
Bare tantalum foil (0.1 mm)	47.9 ± 1.2	5.1	49.5	54.6
Nanoporous Nb_2_O_5_; As formed	25.0 ± 7.0	11.02	56.94	67.96
Nanoporous Nb_2_O_5_; Annealed (10 min at 290 °C)	15.5 ± 1.4	18.11	52.20	70.31
Bare niobium foil (0.05 mm)	61.0 ± 0.0	6.44	34.94	41.38
ZrO_2_ Nanotube; As formed	17.0 ± 1.0	11.56	60.04	71.60
ZrO_2_ Nanotube; Annealed (10 min at 290 °C)	8.5 ± 1.0	12.84	60.77	73.61
Bare zirconium foil (0.05 mm)	58.7 ± 1.3	5.82	38.042	43.87

Figure 3e shows the effect of ZrO_2_ and TiO_2_ nanotube formation with respect to the roughness, *S*_a_, of the bare metals. The presence of the nanotubular ZrO_2_ has increased the roughness of the bare metal and also revealed a higher roughness than the nanotubular layer on the TiO_2_ nanotubes surface with almost the same distribution of *D_i_* (under the conditions of $C_{F}^{-}$ = 0.5 wt %, 10 V). The *S*_skw_ of the ZrO_2_ nanotubular layer is lower than zero, which signifies deep valleys, such as scratches, with a leptokurtic distribution. The surface area and volume indices of ZrO_2_ nanotube are shown in Table 1, along with their roughness amplitude parameters.

Modification of the hydrophilic properties of bare zirconium and titanium after fabrication of nanotubular layer on their surfaces is shown in Figure 3f. Although both nanotubular layers fabricated on the zirconium and titanium surfaces are hydrophilic, the ZrO_2_ nanotubes exhibited a lower water contact angle in comparison to TiO_2_ with nearly the same distribution of *D_i_* ($C_{F}^{-}$ = 0.5 wt %, 10 V), which led to a high surface energy. The water contact angle and surface energy is detailed in Table 2 for ZrO_2_ nanotubes. A direct relationship between roughness and the water contact angle can be observed in Figure 3f.

**Figure 5:**
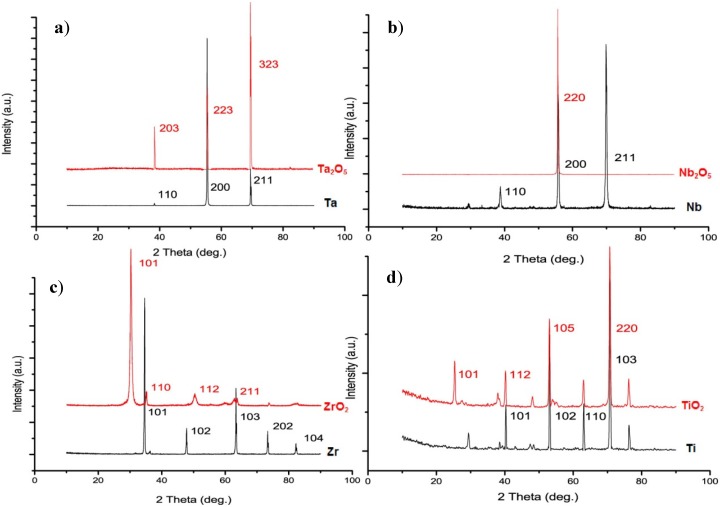
XRD patterns of (**a**) the nanoporous Ta_2_O_5_ and bare tantalum foil; (**b**) the nanoporous Nb_2_O_5_ and bare niobium foil; (**c**) the ZrO_2_ nanotubes and bare zirconium foil; and (**d**) the TiO_2_ nanotubes and bare titanium foil.

Nanoporous Ta_2_O_5_ with a mixture of amorphous and hexagonal phase was fabricated via anodization under the conditions of 1 M H_2_SO_4_ + 3.3 wt % NH_4_F, 20 V for 120 min. The amorphous nanoporous Ta_2_O_5_ transformed into hexagonal Ta_2_O_5_ after annealing at 290 °C for 10 min, as indicated by the XRD patterns in Figure 5a. After annealing the pore size decreased to within the range of 23–49 nm as a result of completion of crystallization. Annealing increased the hydrophilic properties of nanoporous Ta_2_O_5_ layer but as much as TiO_2_ nanotubes because of their different porosity due to their structures.

Nanoporous Nb_2_O_5_ with a mixture of amorphous and monoclinic phase was fabricated via anodization under the conditions of 1 M H_2_SO_4_ + 3.3 wt % NH_4_F, 20 V for 16 min. The amorphous nanoporous Nb_2_O_5_ transformed into base-centered monoclinic Nb_2_O_5_ after annealing at 290 °C for 10 min, as indicated by the XRD patterns in Figure 5b. The pore size, after annealing, was irregular and laid in the range of 26–60 nm. The pore size was determined by the crystallization process during the heat treatment. Annealing did not influence the roughness of the nanoporous Nb_2_O_5_ but decreased the water contact angle of the layer.

**Figure 6:**
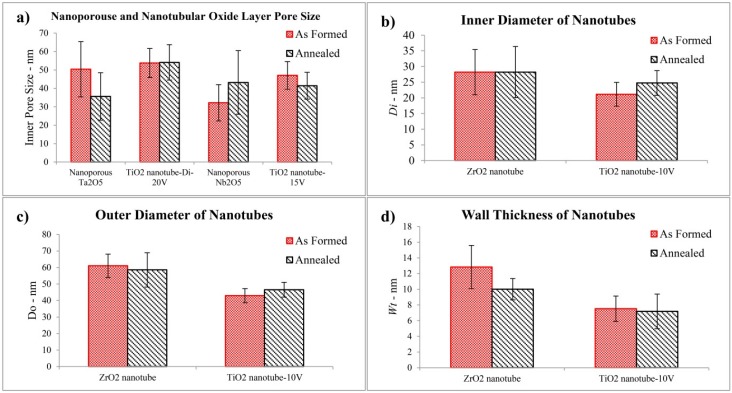
Illustration of pore size of the nanoporous and nanotubular layer.

The cubic ZrO_2_ nanotube was fabricated via anodization under the conditions of 1 M (NH_4_)_2_SO_4_ + 0.3 wt % NH_4_F (with the addition of H_2_SO_4_ to attain a pH = 5) at 30 V for 95 min, as indicated by the XRD patterns in Figure 5c. After annealing, the inner diameter of the nanotubes laid in the range of 20–36 nm, whilst the nanotube outer diameter (*D*_o_) was 48–68 nm and the wall thickness (*W*_t_) was in the range of 9–11 nm. The roughness (*S*_a_) and hydrophilic properties of ZrO_2_ nanotubes increased after annealing. Figure 6a shows the pore sizes of nanoporous Ta_2_O_5_ and Nb_2_O_5_, compared to TiO_2_ nanotube with nearly the same *D*_i_. Figure 6b,c,d shows the *D*_i_, *D*_o_ and *W*_t_ of ZrO_2_ nanotubes compared to TiO_2_ nanotube with nearly the same pore sizes as a visual illustration.

### 2.3. Bioactivity of Nanoporous and Nanotubular Metal Oxide Layer

**Figure 7:**
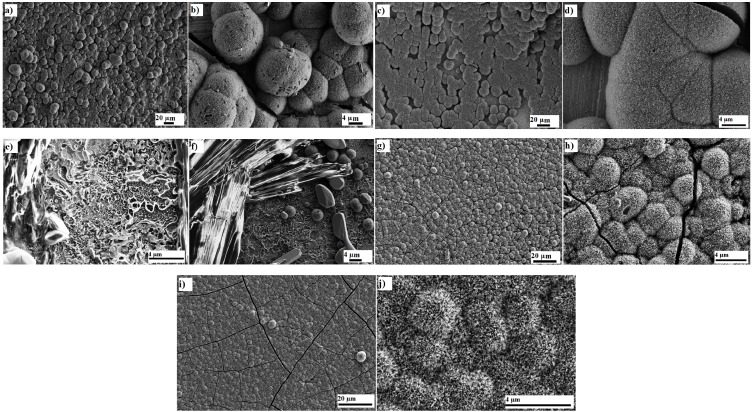
SEM images with (**a**,**e**,**g**) low and (**b**,**h**) high magnification of HA on as formed nanoporous Ta_2_O_5_, Nb_2_O_5_ and ZrO_2_; and (**c**,**i**) low and (**d**,**f**,**j**) high magnification of hydroxyapatite (HA) on annealed nanoporous Ta_2_O_5_, Nb_2_O_5_ and ZrO_2_.

**Figure 8:**
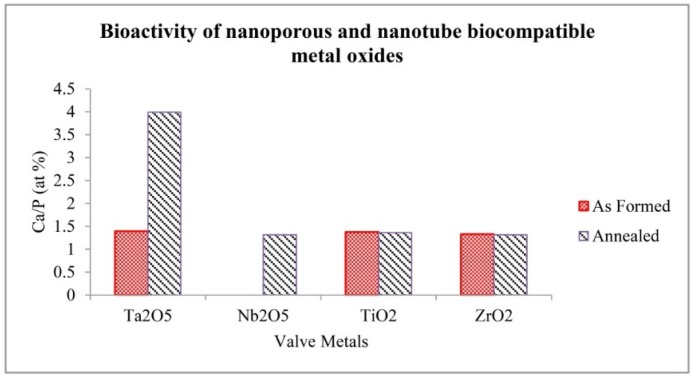
Bioactivity of biocompatible nanoporous and nanotubular oxide metals after 3 weeks in m-SBF at 37 °C.

Artificial implant materials upon implantation *in vivo* are encapsulated by fibrous tissues that isolate them from the surrounding bone. This is not the ideal healing mechanism, whereas a bioactive material is preferred which bonds to living bone by forming a carbonated apatite layer on their surfaces similar to hydroxyapatite (HA) of bone composition [48]. The bioactivity of the biocompatible metals with the as-formed and annealed conditions of the nanoporous and nanotubular metal oxides was assessed by immersion in the m-SBF for a period up to three weeks. The response of the oxide layers to the m-SBF immersion was observed after one day and three weeks. After one-day immersion, no growth of HA was detectable on the surfaces of the as-formed and annealed nanoporous Ta_2_O_5_ and Nb_2_O_5_ and the nanotubular TiO_2_ and ZrO_2_. However, after three weeks immersion as shown in Figure 7, HA were deposited onto the surfaces of the nanoporous and nanotubular layers. The atomic ratio of calcium to phosphate calculated using EDS results after three weeks is indicated in Figure 8.

The M–OH groups located on the surface of biocompatible metal oxides are favored sites for apatite nucleation [48,49,50,51]. First, Ca^2+^ ions are absorbed onto the hydrolyzed nanoporous and nanotubular oxide surface by Coulomb attraction forces. Then, existing phosphate groups inside the m-SBF are adsorbed to the positively charged surface, resulting in the formation of calcium phosphate. The stoichiometric Ca/P (at.%) of octacalcium phosphate [Ca_8_H_2_(PO_4_)_6_ × 5H_2_O], tricalcium phosphate [Ca_3_(PO_4_)_2_] and hydroxyapatite [Ca_10_(PO_4_)_6_(OH)_2_] are 1.33, 1.5 and 1.67, respectively. According to the obtained Ca/P ratio for the metal oxides, annealed nanoporous Ta_2_O_5_ had a high value of Ca/P ratio, which may indicate the presence of a mixture of calcium and phosphate in abovementioned compositions_._ Other nanoporous and nanotubular metal oxide layers induce calcium phosphate and need more time or pre-treatment to induce crystalline hydroxyapatite. Ta_2_O_5_ reaches an isoelectric point at a pH of 2.7–3.0, which can be compared to Nb_2_O_5_ that attains an isoelectric point at a pH of 4.0 [52,53]. Thus, the Ta_2_O_5_ surface, compared to the Nb_2_O_5_ surface, becomes more negatively charged in m-SBF at a pH of 7.4. Therefore there is an appropriate chemical environment for a higher Ca/P atomic ratio to be attained for Ta_2_O_5_ in comparison to Nb_2_O_5_. After annealing, the hydrophilic properties and surface energy of both nanoporous Ta_2_O_5_ and Nb_2_O_5_ increased along with no considerable change on their roughness. It can be concluded that a direct link exists between the hydrophilic properties and the completing crystalline phase of nanoporous layer for inducing the formation and growth of HA. The lower value of *S*_ku_ of the nanoporous Nb_2_O_5_ plus low tendency to form Nb–OH, because of its isoelectric point, made the as-formed nanoporous layer non-bioactive in this study. In addition, the isoelectric point of TiO_2_ has been reported at a pH of 3.9 and the corresponding value for ZrO_2_ is 5.5 [52,53], which would be expected to lead to a higher Ca/P ratio for TiO_2_. After annealing, the hydrophilic properties and surface energy of both ZrO_2_ and TiO_2_ nanotubes increased along with the roughness. The roughness of the nanotubular layer and capillary force of nanotubes influenced the initial reaction sites for apatite nucleation and formation.

## 3. Experimental Section 

### 3.1. Substrate Preparation

Tantalum, 99.95% (10 mm × 10 mm × 0.1 mm), niobium, 99.95% (10 mm × 10 mm × 0.05 mm), zirconium, 99.60% (10 mm ×10 mm × 0.05 mm) and titanium, 99.95% (10 mm ×10 mm ×0.05 mm) foils (Baoji Boxin Metal Materials Co. Ltd., Shaanxi, China) were degreased by sonication in methanol, isopropanol, acetone and ethanol for 15 min each, progressively. The substrates were then washed with deionized water and dried using a nitrogen gas stream. Two electrode configurations were employed using a DC power supply with a 1 cm^2^ platinum plate acting as the counter electrode and placed 4 cm from the working electrode. Electrochemical experiments were performed at room temperature with the electrolyte composed of 1 M H_2_SO_4_ + 3.3 wt % NH_4_F for tantalum and niobium at 20 V for 120 min and 16 min, respectively; 1 M (NH_4_)_2_SO_4_ + 0.3 wt % NH_4_F (with the addition of H_2_SO_4_ to attain pH 5) for zirconium at 30 V for 95 min and 1 M H_2_SO_4_ + 0.5 wt % NH_4_F for titanium at 10, 15 and 20V for 120 min. The electrolytes were prepared from reagent grade chemicals (Sigma Aldridge, Castle Hill, Australia) and deionized water. After the electrochemical treatment, the samples were rinsed with deionized water for 5 min and dried with a nitrogen stream. Annealing of the samples was performed in air at 290 °C for 10 min in a conventional muffle furnace (Nabertherm LT15/13/P330; Nabertherm GmbH, Lilienthal, Germany).

### 3.2. Surface Characterization

Metallographic characterization of the samples was carried out using a field-emission scanning electron microscope (FESEM, ZEISS SUPRA 40 VP, Los Angeles, CA, USA). The characterization of the phase structures was performed by means of XRD using Cu Kα incident radiation at 40 kV and 40 mA (Bruker D8; Bruker Pte Ltd., Singapore). The diffraction patterns were recorded over a 2θ range from 10° to 90° scanned at a step size of 0.02°. For the nanoporous Ta_2_O_5_ and Nb_2_O_5_ layers, the films were scratched off and characterized using XRD.

Roughness parameters were measured using a 3D-Profilometer (Bruker, Contour GT-K1; Bruker Pte Ltd., Singapore) and analyzed using the SurfVision software (Veeco Instruments Inc.; Plainview, NY, USA). Surface topography was characterized by four parameters. Mean Roughness (*S*_a_) and Root Mean Square (RMS) Roughness (*S*_q_) were used to evaluate the vertical characteristics of surface deviation over the 3D surface [54]. The third central moment of profile amplitude, skewness of a 3D surface texture (*S*_skw_) was used to assess the dominant nature of topography. Thus, a value of *S*_skw_ > 0 indicates high peaks about the mean plane and *S*_skw_ < 0 indicates deep valleys such as would be formed from scratches [54]. The sharpness of the height distribution was indicated by a fourth central moment of profile amplitude; that is the kurtosis (*S*_ku_) of the 3D surface texture where *S*_ku_ = 3.0 for a normal distribution of heights. However, when a few high peaks are spread out over the 3D surface, then the distribution is defined as “platykurtic” and *S*_ku_ < 3.0; whereas *S*_ku_ > 3.0 in the instance of the surface exhibiting a high proportion of high peaks and low valleys and this distribution is described as being “leptokurtic” [54].

The water contact angle was measured using a goniometer (NRL C.A. Goniometer, Ramé-hart, Inc.; Succasunna, NJ, USA). Surface energy is calculated based on Owens-Wendt (OW) method [55], as in the following equation:

(1) $$\left(\text{1+cosθ}\right)\gamma_{L}=2\left(\sqrt{\gamma_{L}^{d}\gamma_{S}^{d}}+\sqrt{\gamma_{L}^{p}\gamma_{S}^{p}}\right)$$

where $\gamma_{L}$ is the liquid surface tension (water = 72.8 mJ·m^−2^, glycerol = 63.4 mJ·m^−2^); $\gamma_{L}^{d}$ and $\gamma_{S}^{d}$ are the liquid and solid dispersive component (water =21.8 mJ·m^−2^, glycerol = 37.0 mJ·m^−2^); $\gamma_{L}^{p}$ and $\gamma_{S}^{p}$ are the liquid and solid polar component (water = 51.0 mJ·m^−2^, glycerol = 26.4 mJ·m^−2^), and $\gamma_{S}$ is the sum of $\gamma_{S}^{d}$ and $\gamma_{S}^{p}$ [56].

Bioactivity assessments were carried out by soaking the as-formed and annealed metal oxides samples in modified simulated body fluid (m-SBF) and incubating them at 37 °C for 1 day and 3 weeks. A modified simulated body (m-SBF) [57] with an ion composition nearly equal to blood plasma was prepared by dissolving 5.403 g NaCl, 0.504 g NaHCO_3_, 0.426 g Na_2_CO_3_, 0.225 g KCl, 0.230 g K_2_HPO_4_.3H_2_O, 0.311 g MgCl_2_.6H_2_O, 0.293 g CaCl_2_, and 0.072 g Na_2_SO_4_ in deionized water as previously reported [57]. The m-SBF was buffered at pH 7.4 at 37 °C using 2-(4-(2-hydroxyethyl)-1-piperazinyl) ethane sulfonic acid (aka HEPES) and 1 M NaOH. An amount of 17.892 g HEPES was dissolved in 100 mL 0.2 M NaOH. The ability of the nanostructural surfaces to form apatite was evaluated in a static m-SBF environment. The samples were removed after incubation for 1 day and 3 weeks in m-SBF; then rinsed with deionized water and dried for 24 h at room temperature. The atomic percentage of calcium to phosphorous (Ca/P) ratio was calculated using EDS results.

The nano size distribution measurements were generated from 100 nano-pores or nanotubes at different positions for each sample using the line tool of ImageJ software. An average of three readings per sample was acquired for the roughness parameters, water contact and surface energy measurements.

## 4. Conclusions 

Nanoporous oxide layers on the surfaces of tantalum and niobium and nanotubular layers on the surfaces of zirconium and titanium were fabricated via anodization. The bioactivity of the nanoporous and nanotubular layers was evaluated. The prime conclusions are as follows.

1. The nanoporous Ta_2_O_5_ layer and nanotubular ZrO_2_ and TiO_2_ layer exhibited a higher roughness than their respective bare metals but the nanoporous Nb_2_O_5_ layer exhibited a lower roughness than its bare metal.
2. The nanoporous layers of Ta_2_O_5_ and Nb_2_O_5_ and the nanotubular layers of ZrO_2_ and TiO_2_ revealed an increase in hydrophilic property and surface energy compared to their respective bare metals.
3. The hydrophilic property and the surface energy of the nanoporous layers of Ta_2_O_5_ and Nb_2_O_5_ and the nanotubular layers of ZrO_2_ and TiO_2_ were increased after annealing.
4. After annealing the pore size of nanoporous Ta_2_O_5_ decreased, whereas the pore size of nanoporous Nb_2_O_5_ increased. This is due to their different crystalline phases before and after annealing which possess different lattice parameters. The inner diameter of ZrO_2_ nanotubes did not show any obvious change after annealing because the crystalline phase did not change, whilst annealing led to an increase in the inner diameter of anatase TiO_2_ nanotubes.
5. As formed Ta_2_O_5_ and ZrO_2_ exhibited a good bioactivity similar to TiO_2_. Annealed Ta_2_O_5_ showed a high level of bioactivity, which is promising for biomedical applications. Nb_2_O_5_ did not show bioactivity before annealing; but was bioactive after annealing.
